# Eukaryal composition and diversity in anaerobic soils influenced by the novel chiral insecticide Paichongding

**DOI:** 10.1186/s13568-018-0590-7

**Published:** 2018-04-18

**Authors:** Xiaolin Zhu, Shaomin Zhou, Jing Guo, Xiyue Zhao, Guanghua Yang, Zhiqiang Cai

**Affiliations:** Laboratory of Applied Microbiology and Biotechnology, School of Pharmaceutical Engineering & Life Science, Changzhou University, Changzhou, 213164 China

**Keywords:** Paichongding, Pyrosequencing, Eukaryal community, Soil eukaryal population, Anaerobic soils

## Abstract

Paichongding (IPP) is a neonicotinoid chiral insecticide with independent intellectual property in China. IPP application can increase crop yield, and also lead to insecticide residue and pollution in soils, which will affect microbial population and community composition in soils. In this study, four different types of soils were employed to inquire into the impact of IPP on eukaryal community and species-group through pyrosequencing of 18S rRNA gene amplicons. Fungal population differed in different soils at different days after IPP treatment (DAT). Eukaryal community species in CK (control check) groups were more rich than that with Paichongding sprayed at 5 DAT, while eukaryal species in CK soils at 60 DAT was relatively slight. Shannon’s H’ analysis indicated fungal species in CK soils were also higher at 5 DAT and relative lower at 60 DAT, except in soil C. There are also differences in the phyla and genus levels of the eukaryotic communities in the soil. After IPP application, the relative abundance of *Nectriaceae* increased 3–4 times in soil C. In soil F, *Phaeosphaeriaceae* increased to 57.3% at 5 DAT. The genus of *Guehomyces*, *Aspergillus* and *Alternaria* increased from 3.1 to 9.7, 1.1 to 4.6, 1.5 to 6.7% in soil H, respectively.

## Introduction

Soil microorganism play the most important roles on soil productivity and fertility, including organic matter decomposing, nutrient cycling, and soil aggregates forming (Six et al. 2004), they can change soil chemical and physical properties. Some bacteria and fungi are responsible for providing nutrients and essential materials, which can promote crop growth (Eo and Park 2016; Esitken et al. 2010). Pesticides are more and more popular in modern agriculture in order to control weeds, kill insects, improve the quality and yield of agricultural products. However, pesticides can enter soils via spraying draft during plant treatment, wash-off from treated foliage, release from seeds (Cycon et al. 2013). Pesticide residue in soil affect microbial community composition in soils by altering their population, enzyme activity and microbial diversity, which perhaps reduce soil fertility (Asad et al. 2017; Cai et al. 2016a, b; Zabaloy et al. 2012; Zhang et al. 2014).

Paichongding (IPP, 1-((6-chloropydidin-3-yl) methyl)-7-methyl-8-nitro-5propoxy-1,2,3,5,6,7-hexahydroimidazo[1,2-α-]-pyridine), is a novel neonicotinoid insecticide with independent intellectual property rights (Cai et al. 2016a, b, c; Fu et al. 2013). It has four stereoisomers (*RR*, *SS*, *RS* and *SR*-IPP) because of its two chiral carbon centers. IPP has higher insecticidal activity (40–50 times) compared to imidacloprid-resistance pests, and also has low toxicity to human. In 2009, IPP production in China has reached 1000 tons and was sprayed for almost 3.3 million hectares (Fu et al. 2013).

Insecticides residue can disturb soil ecosystems, some bacteria and fungi are involved in insecticides and other organic pollutants degradation. Insecticides application and accumulation in environment can affect microbial communities diversity and composition (Asad et al. 2017; Cai et al. 2016a, b; Cycon et al. 2013). Accumulation of pesticide residues has been accelerated with the increase in crop growth. Many reports showed that insecticides have potential risk to the soil biochemical properties and it also changes ecosystems through concentrating along food chain (Zabaloy et al. 2012; Zhang et al. 2014). Hence, there is a need to investigate the effect of new insecticides on microbial population, composition and diversity in soils (Asad et al. 2017; Chen et al. 2017).

Previous studies have emphasized the biodegradation pathway and IPP behavior in soils (Cai et al. 2016c; Wang et al. 2016). The effects on bacterial community and soil enzyme activity of IPP were also studied, several studies have addressed the effect of IPP on bacterial community (Cai et al. 2015b, 2016a, b; Chen et al. 2017); however, little information is available on the impact of IPP on soil fungal population and community. In this study, we used four different soils to understand the impact of IPP on soil fungal population and community in soils.

## Materials and methods

### Chemicals and soil samples

Paichongding (IPP, 1-((6-chloropydidin-3-yl)methyl)-7-methyl-8-nitro-5-propoxy-1,2,3,5,6,7-hexahydroimidazo [1,2-α-]-pyridine, chemical purity 98.3%, formula weight FW 366)was manufactured from Jiangsu Kesheng Company Ltd. Soil samples from different location were sampled and used in this study, which were Paddy field on desalting muddy polder (soil C, GB/T-H2121315), yellow paddy soil (soil H, GB/T-A2111511), yellow loam soil (soil F, GB/T-A2111411) and Huangshi soil (soil J, GB/T-G2511211), respectively. The soils were sampled in crop fields located in Cixi (Zhejiang Province), Jingzhou (Hubei Province), Longquan (Fujian Province) and Changzhou (Jiangsu Province), China. The basic physicochemical characteristics of soils were showed in Table 1.

**Table 1:** Physico-chemical characteristics of the experimental soils

Characteristics	Yellow loam soil (F)	Huangshi soil (J)	Paddy field on desalting muddy polder (C)	Yellow paddy soil (H)
Location	Longquan, Fujian Province	Changzhou, Jiangsu Province	Cixi, Zhejiang Province	Jingzhou, Hubei Province
pH (H_2_O)	6.63	5.95	8.25	6.70
OM, %	2.67	1.52	2.48	1.98
CEC, cmol kg^−1^	14.09	7.11	16.1	13.9
Clay, %	38.7	33.5	24.3	33.0
Silt, %	50.4	49.8	71.1	51.2
Sand, %	10.9	16.7	4.6	15.8
Texture, % (mm)				
< 0.01	67.4	60.7	64.7	67.4
0.01–0.09	28.3	32.6	34.5	28.3
> 0.09	4.3	6.7	0.8	4.3
Total N, %	0.24	0.08	1.03	0.12
P, mg kg^−1^	21.25	7.65	15.37	10.25
K, g kg^−1^	13.47	10.7	22.9	5.22

*OM* organic matter, *CEC* cation exchange capacity

### Experiments procedure

To study the effect of Paichongding on eukaryal community in anaerobic soils, soil sample was separated into two sets: one was pre-incubated for acclimatizing microorganism in soils without IPP inoculation (CK set), and the second was inoculated with 10 mg kg^−1^ of IPP in soil. All the experiments of each soil were carried out in triplicate. The soil moisture content was maintained at about 60%. The incubation temperature was 25 ± 1 °C. Ten grams of soil were collected from the flask to counter fungal population and DNA extraction at 5, 20, 30, 45, 60, 75 and 100 days after treatment (DAT). Soil fungal population counting was assayed through most probable number method (MPN) according with previous reported methods (Cai et al. 2016a, b).

### Soil DNA extraction and pyrosequencing, bioinformatics analysis

Soil DNA extraction was based on the manufacturer’s protocol (FastDNA Spin kit, MP Biomedicals, USA). The total DNA was purified using agarose gel electrophoresis. Pure DNA was extracted using extraction kit (Roche). DNA quality was assessed with a ScanDrop 200 spectrophotometer.

The primers ITS1F (5′-CTTGGTCATTTAGAGGAAGTAA-3′) and ITS2R (5′-GCTGCGTTCTTCATCGATGC-3′) were used to amplify 18S rRNA gene fragment. The PCR reaction program and PCR products purification was according with previous reported methods (Cai et al. 2015a, 2016a). Totally 590,023 18S rRNA sequence reads were filtered, Trimmomatic was applied to denoised and processed. The sequences were analyzed through QIIME. Operational taxonomic units (OTUs) was used at 97% sequence similarity. The methods used for Rarefaction curves, Shannon–Wiener curves, eukaryal communities composition, Venn and PcoA analysis, and eukaryal genus heatmap were in accordance with the previous reported methods (Cai et al. 2015a, 2016a). The metagenomic sequencing data were deposited in the BioProject database (BioProject ID: PRJNA425705).

## Results

### Effect of IPP on soil eukaryal population

**Fig. 1:**
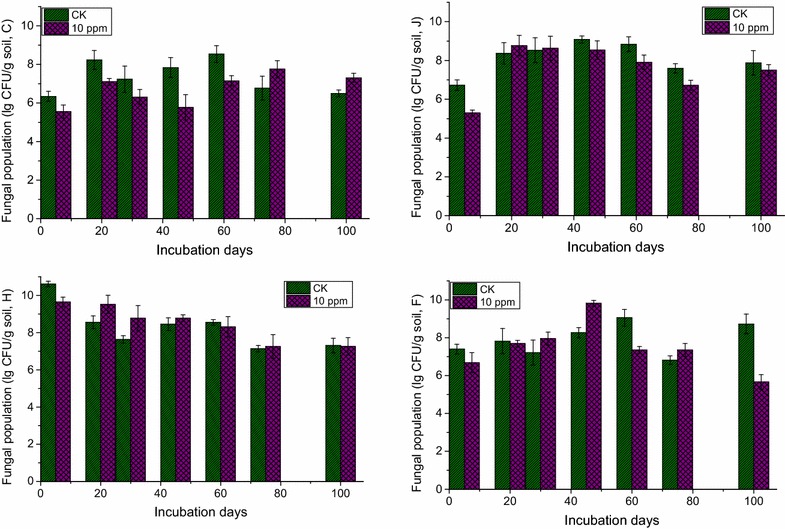
Effect of IPP on soil fungal population for different incubation periods in soils

The effect of IPP on fungal population in anaerobic soils was indicated in Fig. 1. The total number of fungi in soil C decreased significantly before 60 DAT compared with CK group, and then increased at 75 and 100 DAT. In soil F, the total number of fungi increased at 30 and 45 DAT, then decreased significantly at 100 DAT. Fungal population was unchangeable compared with the CK group at 20 and 30 DAT, after 45 DAT the fungal population decreased in soil J. While in soil H, fungal population increased significantly before 45 DAT, and then keep unchangeable compared with the CK group.

### Eukaryal community species diversity and structure

**Fig. 2:**
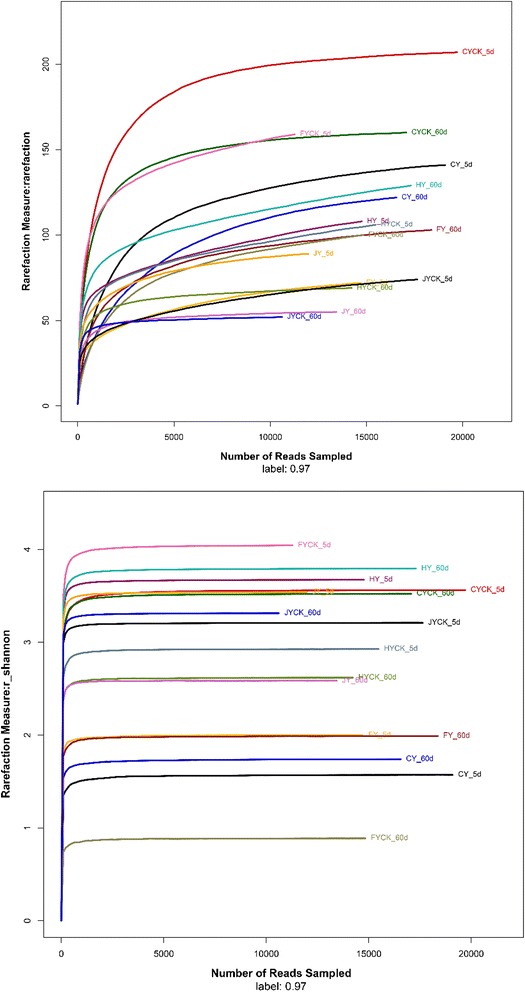
Rarefaction curves (**a**) and Shannon–Wiener curves (**b**) (CYCK: CK of soil C; CY: soil C; FY: soil F; FYCK: CK of soil F; HY: soil H; HYCK: CK of soil H; JY: soil J; JYCK: CK of soil J)

Species diversity of microbial community was shown in Fig. 2a. The results showed that eukaryal community species in CK group have more diverse than that in samples with Paichongding application at 5 DAT, while eukaryal species in CK soils at 60 DAT was relatively lower. Shannon’s H’ analysis indicated fungal species in CK soils were also higher at 5 DAT and relative lower at 60 DAT, except in C soil. The results of rarefaction analysis (Fig. 2a) were also almost the same compared with Shannon’s H’ (Fig. 2b).

**Fig. 3:**
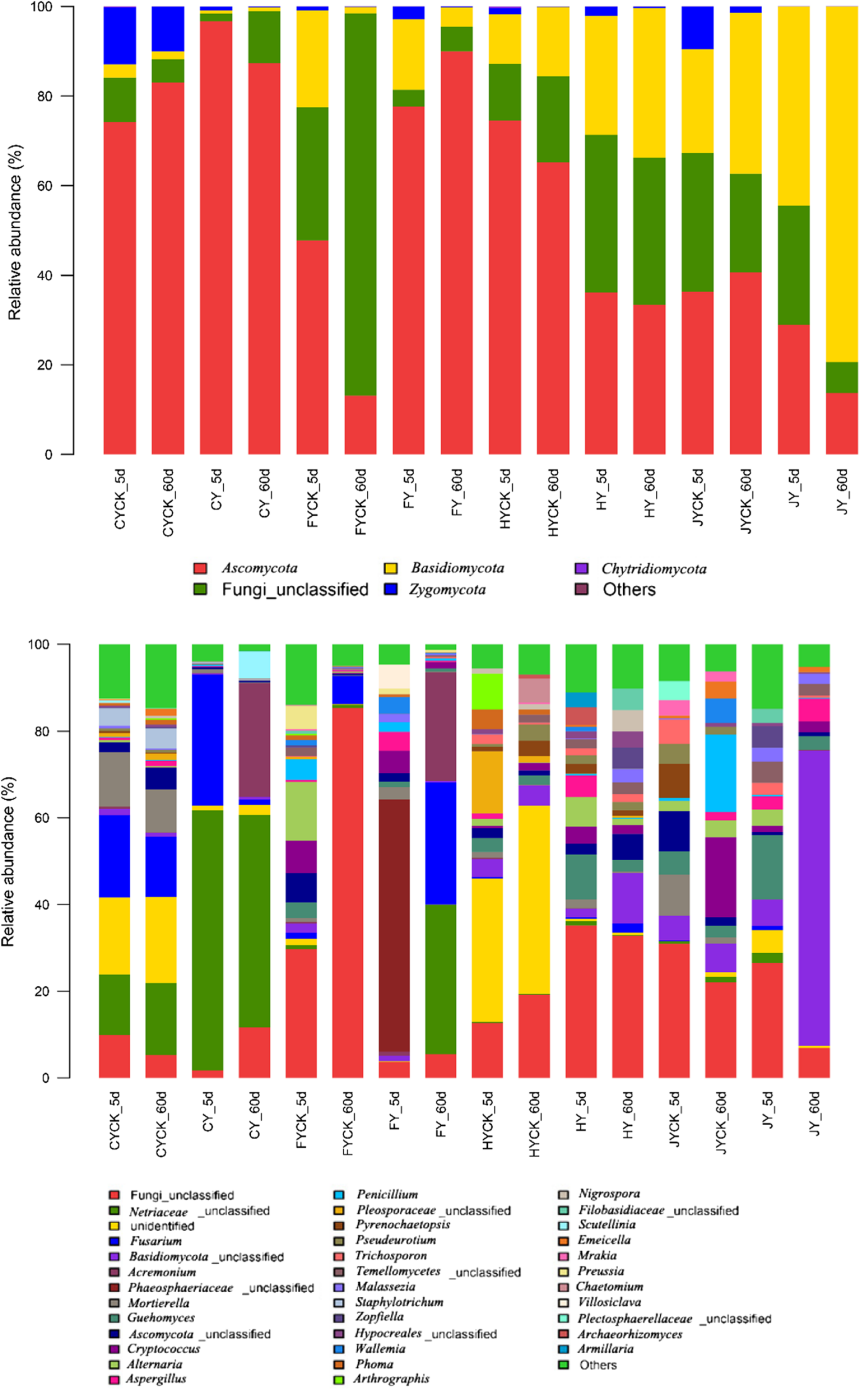
Eukaryal composition of the different communities (**a** Percentage of relative read abundance of eukaryal phyla within each community; **b** Percentage of relative read abundance of eukaryal genus within each community)

All the reads in Fig. 3 were belonged to the family of fungi. *Ascomycota* were the most abundant phylum in all samples (33.9–95.6%, Fig. 5a), apart from soil F without Paichonging sprayed and soil J with IPP sprayed at 5 and 60 DAT. The relative abundance of *Basidiomycota* increased from 26.1 to 33.0% in soil H, from 43.5 to 79.2% in soil J after IPP application. There were four phyla identified in this study, *Ascomycota*, *Basidiomycota*, *Chytridiomycota* and *Zygomycota*. *Zygomycota* was only found in the CK C and H soils, and its abundance was about 0.01–0.03%.

The composition of fungal community is strongly influenced by IPP in crop soils. The most abundant genus were *Nectriaceae*, *Fusarium*, *Mortierella*, *Ascomycota* and *Staphylotrichum* in C soil (CK group), after IPP application in soil C, the relative abundance of *Nectriaceae* increased 3–4 times. The genus of *Fusarium* was also increased significantly at 5 DAT, while decreased sharply at 60 DAT. *Acremonium* reach 4341 reads, which was 30 times compared with CK group (14 reads). *Scutellinia* was only occurred at 60 DAT in C soil after IPP application and reach 1041 reads. In soil F, *Phaeosphaeriaceae* increased to 57.3% at 5 DAT after IPP application, then it disappeared and *Acremonium*, *Fusarium* and *Nectriaceae* occurred and increased significantly at 60 DAT. At 5 DAT in soil H, *Guehomyces*, *Aspergillus* and *Alternaria* increased from 3.1 to 9.7, 1.1 to 4.6, 1.5 to 6.7%, respectively. At 60 DAT, *Guehomyces* decreased again. The genus of *Basidiomycota* had 9142 reads and its relative abundance reached 68.3% at 60 DAT after IPP application.

**Fig. 4:**
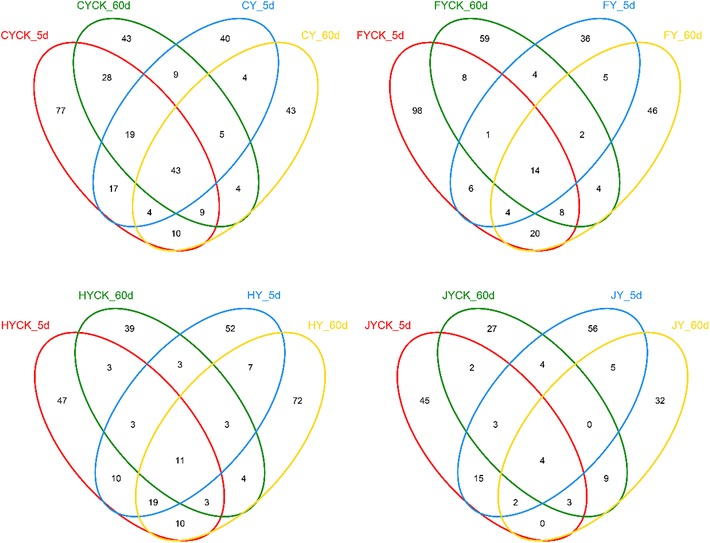
Fungal Venn analysis in soils

The overlaps of community were displayed as three-set Venn diagrams (Fig. 4). Soil C and H shared almost identical number of OTUs. The shared OTUs keep unchangeable after IPP application. The OTUs in soils with IPP sprayed was lower than that in CK soil. After application of IPP, OTUs increased in soil F and J. In soil C, there were 141, 207, 122 and 141 OTUs in CY-5d, CYCK-5d, CY-60d and CYCK-60d soil samples, and they shared 43 same OTUs.

## Discussion

The research results indicated that significant difference of fungal population existed before and after IPP spraying in anaerobic soils. The degradation efficiency and transformation rate of organic pollutants (such as pesticide, germicide and insecticide, etc.) in soils mainly depended on soil microbial population and its activity (Asad et al. 2017; Cycon et al. 2013; Eo and Park 2016; Kuppusamy et al. 2016). The chemical and biological processes in soils can lead to fungal population, including enzymatic reaction and organic pollutants biological behavior etc., response to an ecological disturbance, which depend on the presence of pesticide or insecticide (Asad et al. 2017; Cycon et al. 2013; Eo and Park 2016; Zabaloy et al. 2012). The change of microbial population and soil enzyme activity after insecticide application varied differently in different soils, which lead to hardly predict with certain model, because many inter reactions in soils existed in different conditions. The results indicated that the soil enzyme activity and fungal population were affected after IPP application, soil enzyme activity is one of key factors during nutrient cycling in soils (Asad et al. 2017; Cai et al. 2015a, 2016b). Neonicotinoid insecticides are high toxicity to vertebrates, and its use has been partly restricted for their effects on pollinators (Zeng et al. 2013). This study also suggested that IPP also can inhibit fungal population and has toxicity to fungi, which are in accord with published reports (Cai et al. 2016a, b).

**Fig. 5:**
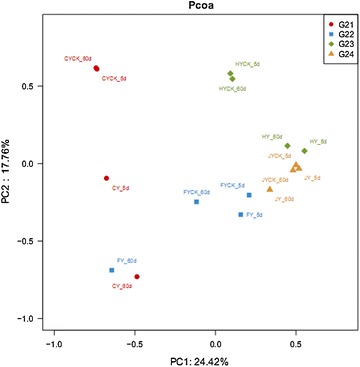
Fungal PCoA analysis results in soils

**Fig. 6:**
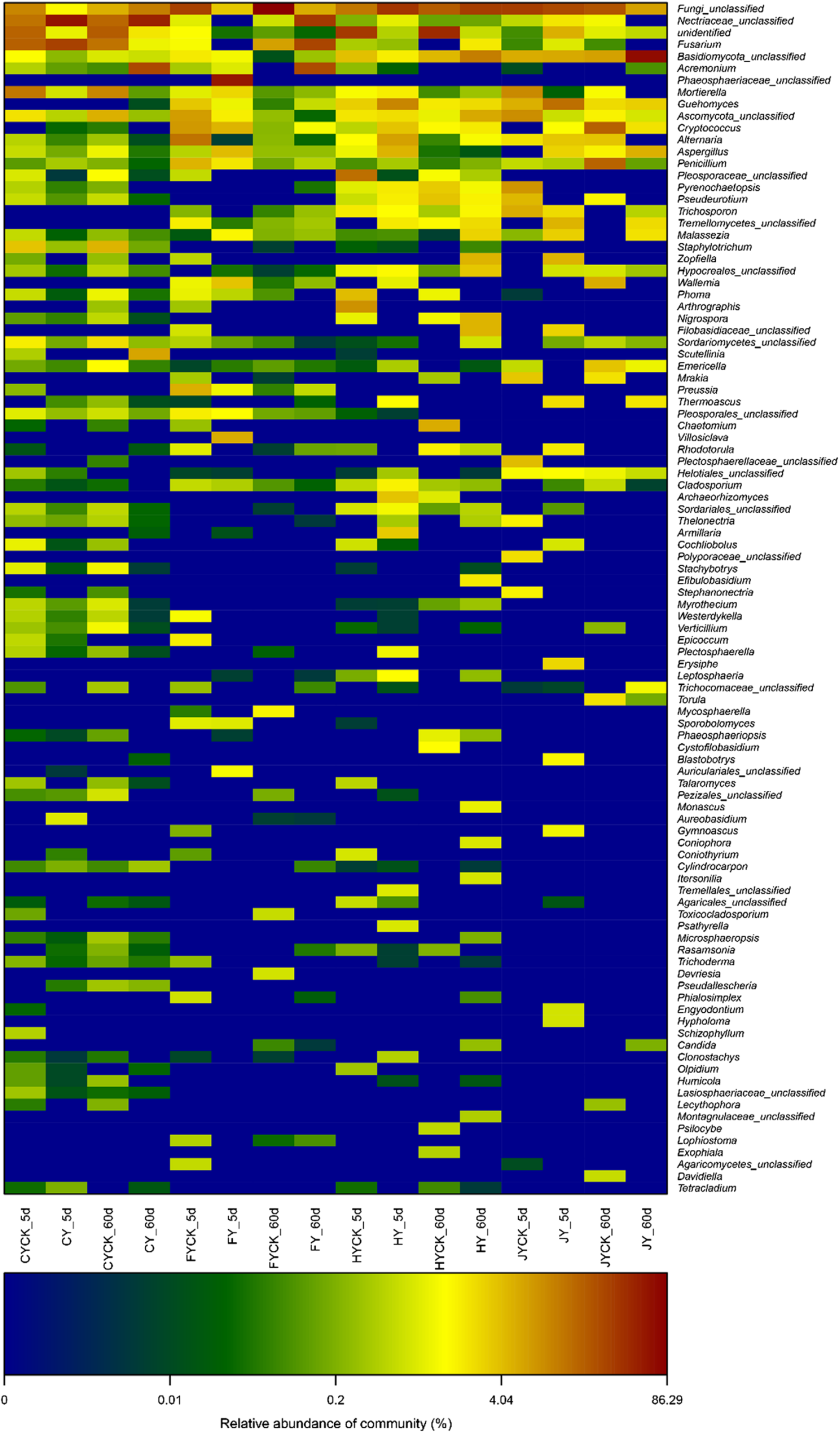
Distribution heatmap of eukaryal genus arranged by hierarchical clustering in soils with different treatment

In this study, we investigated the correlation of the eukaryal community patterns to with IPP sprayed and CK soil sample using the method of conducted the PCoA (Principal Coordinates Analysis), which based on sample similarity matrix from the same soil. It revealed the PCoA results (Fig. 5) that samples may be differentiated after IPP sprayed, whereas samples from same soils were nearly together because they shared similar community structure patterns. The soil fungal community after spraying IPP shared higher similarity according to the clustered heatmap analysis, which based on the eukaryal community at the genus level (Fig. 6). The eukaryal profile in CK soils was distant than that soils after Paichongding sprayed.

The organic matter (OM) content is the key factor during IPP biodegradation in soils. Higher OM content brought about higher IPP degradation rate and higher microbial population (Cai et al. 2016a, b), previous studied also showed the higher TC content can enhance microorganism growth (Hirano et al. 2007; Eo and Park 2016; Chen et al. 2016). Eukaryal community diversity was less diverse after IPP sprayed in soils, Shannon’s H and rarefaction were also relative low, which was with accordance with the results of IPP effect on bacterial community and species.

This study showed the IPP can affect microbial population and community species. IPP could make some of phyla and genus increase or decrease in soils. These variation was presumably due to IPP spraying and properly accumulation of IPP metabolites in soils (Cai et al. 2015a, b, 2016a, b; Fu et al. 2013; Li et al. 2013; Wang et al. 2013). The heatmap also revealed clearly that the eukaryal communities were different in same soil (Fig. 6). Specific eukaryal communities and populations occurred under specific stresses in environment, which indicated that the change of microbial communities composition was caused by specific environmental stresses, such as biodegradation intermediates accumulation, interaction with other organic pollutants in soils.

## Abbreviations

IPP: Paichongding
DAT: days after IPP treatment
CK: control check
soil C: paddy field on desalting muddy polder
soil H: yellow paddy soil
soil F: yellow loam soil
soil J: Huangshi soil
MPN: probable number method
OTUs: operational taxonomic units
OM: organic matter
